# Sirtuins as Players in the Signal Transduction of *Citrus* Flavonoids

**DOI:** 10.3390/ijms25041956

**Published:** 2024-02-06

**Authors:** Giovanni Enrico Lombardo, Caterina Russo, Alessandro Maugeri, Michele Navarra

**Affiliations:** 1 Department of Chemical, Biological, Pharmaceutical and Environmental Sciences, University of Messina, 98166 Messina, Italy; gelombardo@unime.it (G.E.L.); carusso@unime.it (C.R.); mnavarra@unime.it (M.N.); 2 Department of Veterinary Sciences, University of Messina, 98168 Messina, Italy

**Keywords:** SIRT1, SIRT2, SIRT3, SIRT4, SIRT5, SIRT6, SIRT7, polyphenols, flavonoids, *Citrus* fruits

## Abstract

Sirtuins (SIRTs) belong to the family of nicotine adenine dinucleotide (NAD^+^)-dependent class III histone deacetylases, which come into play in the regulation of epigenetic processes through the deacetylation of histones and other substrates. The human genome encodes for seven homologs (SIRT1-7), which are localized into the nucleus, cytoplasm, and mitochondria, with different enzymatic activities and regulatory mechanisms. Indeed, SIRTs are involved in different physio-pathological processes responsible for the onset of several human illnesses, such as cardiovascular and neurodegenerative diseases, obesity and diabetes, age-related disorders, and cancer. Nowadays, it is well-known that *Citrus* fruits, typical of the Mediterranean diet, are an important source of bioactive compounds, such as polyphenols. Among these, flavonoids are recognized as potential agents endowed with a wide range of beneficial properties, including antioxidant, anti-inflammatory, hypolipidemic, and antitumoral ones. On these bases, we offer a comprehensive overview on biological effects exerted by *Citrus* flavonoids via targeting SIRTs, which acted as modulator of several signaling pathways. According to the reported studies, *Citrus* flavonoids appear to be promising SIRT modulators in many different pathologies, a role which might be potentially evaluated in future therapies, along with encouraging the study of those SIRT members which still lack proper evidence on their support.

## 1. Introduction

**Figure 1:**
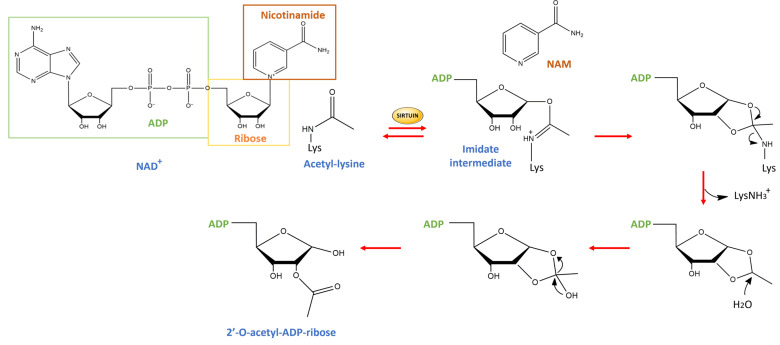
The chemical reaction catalyzed by SIRTs consists of forming an imidate intermediate, which can either combine with nicotinamide, regenerating NAD^+^, or proceed forward until deacetylation of acetyl-lysine residues.

Sirtuins, known as silent information regulator proteins (SIRTs), are nicotine adenine dinucleotide (NAD^+^)-dependent class III histone deacetylases, able to regulate epigenetic processes by removing the acetylated groups from histones and other substrates [1,2]. Indeed, in recent decades, SIRTs emerged for their capability to also target transcription factors and metabolic enzymes, playing a pivotal role in the regulation of cellular homeostasis [3]. The chemical reaction promoted by SIRTs consists initially of the cleavage of the *N*-glycosidic bond of NAD^+^, forming an imidate intermediate, which can either combine with nicotinamide, regenerating NAD^+^, or proceed forward until deacetylation. The latter is favored by the formation of a bicyclic intermediate through the nucleophilic bond between the imidate and the 2′-OH group. Finally, the collapse of the bicyclic intermediate leads to the deacetylated lysine product (Figure 1) [4].

Interestingly, SIRTs are found in all living organisms, phylogenetically conserved in eubacteria, archaea, and eukaryotes [5,6]. In this regard, the first member of this family was the silent information regulator 2 (Sir2p), originally known as mating type regulator 1 protein (MAR1), discovered more than 40 years ago by Karl and collaborators in the budding of *Saccharomyces cerevisiae* [7]. The human genome encodes for seven homologs (SIRT1-7) with different enzymatic activities, regulatory mechanisms, subcellular localizations, and targets [8]. In particular, SIRT1 is localized in the nucleus, where it interacts with several transcription regulator factors, while SIRT2 is predominant in the cytosol, although it can also translocate into the nucleus [5,9]. SIRT3, SIRT4, and SIRT5 are mitochondrial enzymes and play a key role in a wide range of mitochondrial metabolic processes, whereas SIRT6 and SIRT7 are located in the nucleus and nucleolus, respectively [10]. From a structural point of view, human SIRTs are characterized by a common core of about 250 amino acids with N- and C-terminal regions of different length acquiring diverse conformational states, which permit bonds with multiple substrates exerting different features in the cells [11]. 

**Figure 2:**
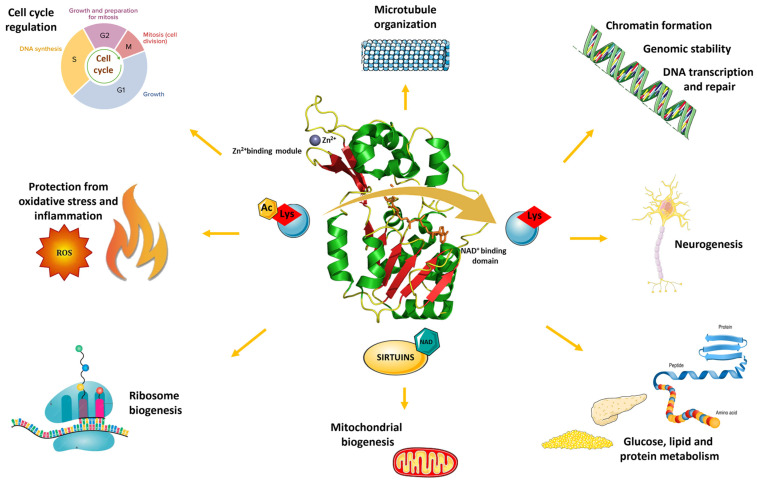
Biological effects mediated by the deacetylase activity of SIRTs.

The SIRT family plays a biological role in the human organism as well as in specific disease cases. Physiologically, SIRTs are implied in several processes which take place within the cell, such as energy metabolism, through regulation of mitochondrial (i.e., SIRT1, SIRT3) and ribosome (SIRT7) biogenesis and maintaining lipid (i.e., SIRT1, SIRT5, and SIRT6), glucose (i.e., SIRT4 and SIRT6), and protein (i.e., SIRT5) homeostasis. Growth, differentiation, and cell death are also subjected to the control of SIRTs, through regulation of the cell cycle (i.e., SIRT2) and chromatin formation (i.e., SIRT1). SIRTs are also able to guarantee genomic stability, to monitor biological processes such as those of DNA transcription and repair, and microtubule organization (i.e., SIRT6) [12]. In addition, mechanisms of neurogenesis and protective effects against oxidative (i.e., SIRT3) and inflammatory (i.e., SIRT6) events depend on SIRT activity as well [13]. On these bases, SIRTs act as “cellular sensors” since, in response to stress phenomena caused by metabolic deficits or oxidative damage, they counteract aging by contributing to cell survival [14] (Figure 2). Consistent with this, changes in SIRT expression and activity are associated with pathological conditions, ranging from insulin resistance and type 2 diabetes (T2D) [15], to oxidative stress and kidney damage [16], cardiovascular and gynecological diseases [2,17,18], rheumatoid arthritis [19], neuro-inflammation [20,21], and various types of cancer [22,23,24]. In this light, a regulation of SIRT activity might support the treatment of the above-mentioned diseases.

Nowadays, we are observing an ever-increasing interest in phytochemicals from several plant sources. This is due mainly to their beneficial effects in counteracting a wide plethora of illnesses such as obesity and its related comorbidities [25,26], inflammation [27,28], cancer [29,30] and neurodegeneration [31,32]. Phytochemicals are organic metabolites produced by plants and fungi and can be classified in two groups according to their functions. The first group includes primary metabolites involved in plant growth, development, reproduction, and metabolism, while the second one consists of secondary metabolites, which are able to protect plants from injury and diseases [33]. Among the latter, flavonoids are largely studied for their capability to protect and mitigate several diseases, exerting many different biological effects such as cardio-protective [34], anti-cancer [35,36], neuroprotective [37], antioxidant [38], and anti-obesity effects, as well as their role in the management of insulin resistance [39,40]. Interestingly, several scientific reports highlighted the beneficial effects of flavonoids contained in both *Citrus* fruits and their juices that, together with their byproducts [41], represent a real treasure for human health [42,43], for their capability to target multiple molecular targets [44,45,46,47]. 

This review collects the most relevant evidence on the potential of *Citrus* flavonoids to target and modulate human SIRTs, thus shedding light on the key role of these proteins in several physio-pathological processes for eventual future therapeutic approaches.

## 2. *Citrus* Fruits and their Flavonoids

**Figure 3:**
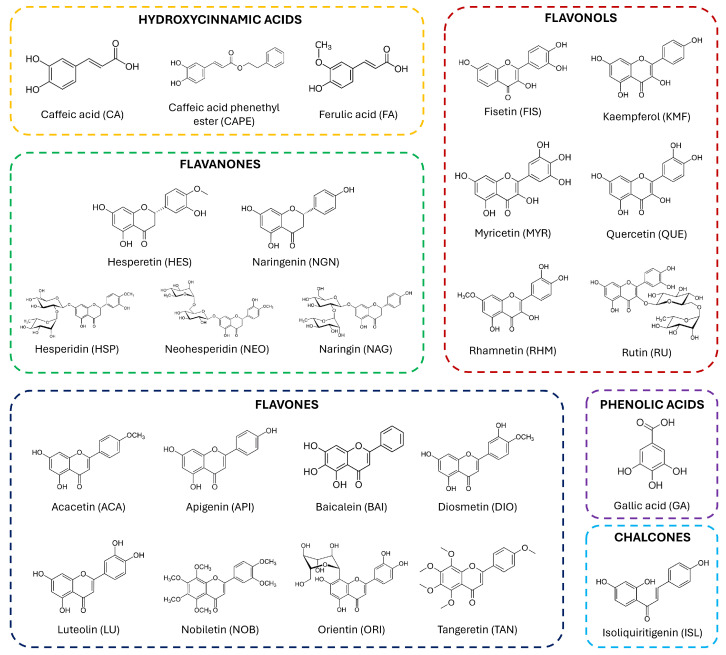
Molecular structures of the main flavonoids and polyphenolic precursors present in *Citrus* fruits and investigated for their role in the various SIRTs.

*Citrus* fruits are typical of the Mediterranean diet and represent one of the pillars of many other dietary patterns. According to several studies, *Citrus* derivatives play a pivotal role in the prevention and/or management of different diseases [48,49,50]. The actors of these effects are acknowledged to be the flavonoids present in *Citrus* fruits. The biosynthesis of these compounds arises from the oxidative deamination of the aromatic amino acid phenylalanine and tyrosine in plants. The originated coumaroyl derivative can either undergo oxidation to give molecules such as caffeic and ferulic acids, or condensate with hydroxybenzyl derivatives (i.e., gallic acid), coming from the shikimate pathway, to give chalcones. The cyclization of these latter compounds creates the backbone of flavonoids, which is a benzo-pyrone moiety. Depending on the presence of unsaturation in the pyrone ring, flavonoids may be divided into flavones and flavanones [38]. In addition, the presence of a hydroxy group in the pyrone ring characterizes two other subclasses, namely flavonols (3-hydroxyflavones) and flavanonols (3-hydroxyflavanone). The most representative ones in *Citrus* fruits are flavones, flavanones, and flavonols (Figure 3). This basic structure can be variously substituted with both hydroxy and methoxy groups. In particular, in *Citrus* fruits, polyhydroxy flavonoids are generally present in juice and pulp, while polymethoxy flavonoids are present in the peel, and hence in the essential oils [51]. The aglycones can be also linked to sugar residues, which are commonly D-glucose and L-rhamnose in *Citrus* fruits, via the hydroxy groups (O-glycosides) or, less commonly for *Citrus* fruits, via the carbons of the benzopyrone moiety (C-glycosides).

Among the *Citrus* species mainly cultivated, *Citrus sinensis* (orange) represents the most relevant fruit crop worldwide. An interesting anti-anxiety property has been ascribed to the essential oil of this fruit [52]. It has been reported that orange juice intake improves lipid metabolism by reducing triglycerides and cholesterol levels in obese and insulin-resistant subjects [53]. Interestingly, it is the flavonoid content in orange juice that is crucial in its effects. Indeed, several studies reported that the flavonoid-rich extract from orange juice (OJe) exerts, among other things, anti-inflammatory [54] and anti-convulsant [55] effects. 

*Citrus limon* (lemon) is the other uncontested member of the *Citrus* genus and several studies report its beneficial effects on human health [56]. Indeed, it has been shown that in lemon juice nanovesicles, plenty of flavonoids hampered the proliferation of different tumor cell lines by activating TRAIL-mediated apoptotic cell death [57]. These flavonoid-rich nanovesicles have been also shown to inhibit redox imbalance in H_2_O_2_-stressed human dermal fibroblasts, via the AhR/nuclear transcription factor 2 (Nrf2) signaling pathway, as well as in LPS-stressed zebrafish [58].

Other scientific reports investigated the beneficial effects of *Citrus reticulata* (mandarin) juice (MJ) in both in vitro and in vivo experimental models. In particular, Testai and collaborators highlighted MJ capability to counteract metabolic syndrome, improving mitochondrial membrane potential in high-fat diet-fed rats [59]. On the other hand, it has been found that MJ is able to restore mitochondrial membrane potential, exerting antioxidant effects [60], as well as to hamper the proliferation and migration of anaplastic thyroid cancer cells [61]. 

*Citrus bergamia* Risso (bergamot) is cultivated to retrieve its essential oil (BEO), mainly employed in the perfume industry and aromatherapy [62]. Moreover, it has been found that BEO is able to exert anti-inflammatory and analgesic properties [63], while its coumarin fraction at low concentration hinders cancer cell proliferation [64]. Bergamot juice (BJ), which was considered as an industrial byproduct until the last decade, has been recently considered together with its flavonoid-rich extract (BJe), for its anti-inflammatory [65], anticancer [66,67], and anti-infective [68,69] properties, and in association with resveratrol and curcumin, it was shown to be able to mitigate cadmium-induced kidney damage [70]. Furthermore, recent studies highlighted that bergamot flavonoid fraction can be employed in the management of metabolic syndrome and against non-alcoholic fatty liver diseases (NAFLDs) [71,72].

## 3. SIRT1

**Figure 4:**
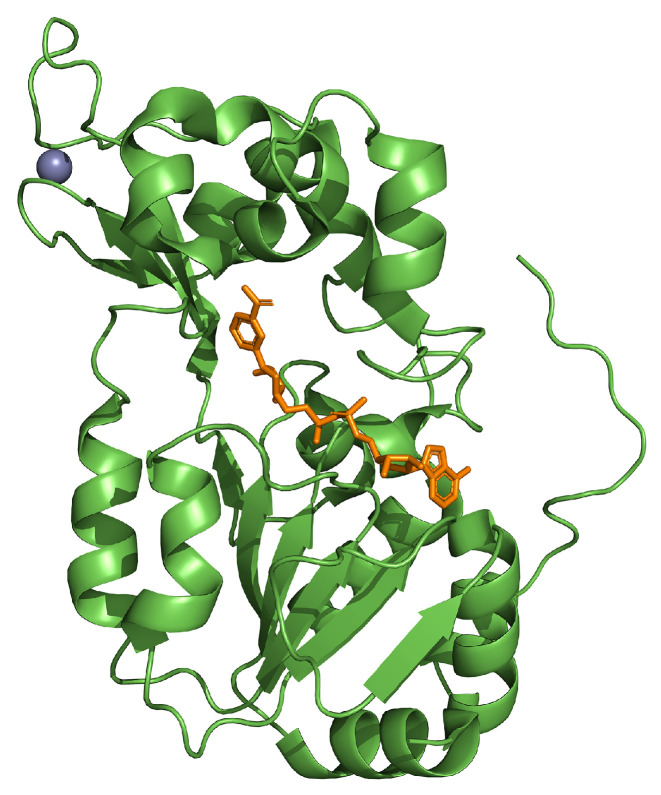
Crystal structure of the SIRT1 catalytic domain (in green) bound to NAD (in orange) and zinc (in violet; PDB: 4I5I).

SIRT1 is found in the cellular nucleus, and it is encoded by the *SIRT1* gene located on chromosome 10q22.1. It is characterized by a catalytic core containing a fold with a larger NAD^+^ binding sub-domain of Rossman and a smaller subdomain containing a Zn^2+^ binding site [73] (Figure 4). Moreover, SIRT1 is able to deacetylate histones (H1, H3, and H4) and transcriptional factors such as p53 and NF-κB by employing NAD^+^. This mechanism permits the attachment of ADP-ribose with the acetylic moiety of the substrate, releasing nicotinamide (NAM) and 2′-O-acetyl-adenosine diphosphate-ribose [74].

Several studies reported the involvement of SIRT1 in the pathogenesis, development, and treatment of different illnesses, including inflammation [75], cancer [74], and neurological and metabolic diseases [76,77,78]. The modulation of SIRT1 is one of the multiple mechanisms by which *Citrus* flavonoids exert their biological properties. 

In the context of oxidative pathogenesis, flavonoids were shown to exert a positive modulation on SIRT1. Indeed, in oxidative stress conditions such as those caused by exposure to environmental contaminants, Helmy and co-workers demonstrated that hesperidin (HES) exerts antioxidant effects through SIRT1 activation. This counteracted the aberration of miR-126-3p and miR-181a observed in testicular damage and promoted their expression [79]. In addition, the flavonoid fisetin (FIS) was able to improve the quality of sperm in Wistar rats, by counteracting oxidative toxicity induced by glutamate at the testicular level [80], via an increase in SIRT1 and p-AMPK. Similarly, a common *Citrus* flavonoid, naringenin (NGN), was able to activate the AMPKα/SIRT1 axis, thus restoring mitochondrial Ca^2+^ balance and lowering radical oxygen species (ROS) levels in in vitro and in vivo models of ROS-induced endothelial damage [81]. Consistently, the same flavonoid protected against pain sensitivity caused by chronically disturbed sleep, through the activation of SIRT1, hampering both oxidative stress and inflammation [82].

Given the well-known link between inflammation and oxidative stress, it has been reported that FIS and the quercetin (QUE) glycoside, rutin (RU), mitigated both inflammation and oxidative stress in nucleus pulposus of mesenchymal stem cells (NPMSCs) and in chondrocytes, respectively, by activating SIRT1 [83,84]. Along the same line, an increase in SIRT1 deacetylase activity mediated by myricetin (MYR) was associated with NF-κB inhibition in A549 cells according to an in vitro model of chronic obstructive pulmonary diseases and asthma [85]. This flavonoid has been largely studied for its antioxidant, antifungal [86], antiviral [87], neuroprotective [88], and anticancer [89] properties. Interestingly, Wang and colleagues proved that HES was capable of counteracting both inflammation and oxidative stress, via SIRT1/peroxisome proliferator-activated receptor-gamma coactivator 1-alpha (PGC-1α)/NF-κB signaling pathways [90]. Remarkably, through the same mechanism of action, NGN was able to regulate ovarian function in polycystic ovary syndrome [91]. Another molecular mechanism underlying the anti-inflammatory and antioxidant effects of *Citrus* flavonoids leading to SIRT1 upregulation was investigated by Abo El-Magd. In this study, HES was able to hamper both inflammation and oxidative stress through the activation of the FOXO/SIRT1 axis in a murine model of hepatic encephalopathy [92]. Interestingly, the flavone luteolin (LU), largely found in *Citrus* fruits, revealed its capability to hamper renal fibrosis acting on the same SIRT1/FOXO3 pathway [93].

Several studies supported the involvement of the AMPK/SIRT1 axis in the anti-inflammatory effects observed for flavonoids. Remarkably, Risitano and co-workers reported that BJe was able to mitigate the inflammatory response in LPS-stimulated THP-1 cells, through SIRT1-mediated NF-κB inhibition [94]. Interestingly, molecular mechanisms underlying the anti-inflammatory effect of Bje were deeply investigated by Maugeri and colleagues. This study reported that this extract and its single flavonoids are direct activators of SIRT1 in both cell-free and in silico experimental models, and it was able to increase SIRT1 deacetylase activity by a mechanism implying 5′ adenosine monophosphate-activated protein kinase (AMPK) activation in vitro [95].

This mechanism was common to the other two in vitro studies. Indeed, naringin (NAR) and hesperetin (HSP) exerted their anti-inflammatory effects in human nucleus pulposus cells (NPCs) [96] and hepatocellular carcinoma (HepG2) cell lines [97], via activation of the SIRT1/AMPK axis. Again, this activation was favored by other *Citrus* flavonoids, including LU, orientin (ORI), and tangeretin (TAN). In this way, LU prevented atherosclerosis in LDL receptor-deficient mice by reducing macrophage inflammation [98], while ORI was able to mitigate mitochondrial dysfunction in rat NPCs [99]. Finally, TAN showed both anti-inflammatory and antioxidant properties in an in vitro model of neuroprotection, promoting both the upregulation of SIRT1 and the phosphorylation of AMPK, which hindered NF-κB activation [100]. Similarly, caffeic acid (CA) and its phenethyl ester (CAPE), activating the AMPK/SIRT1 axis, showed neuroprotective effects against Cd-induced neurotoxicity, by attenuating neuronal apoptosis and neuro-inflammation [101,102]. Therefore, the activation of AMPKα/SIRT1 pathways can be considered as one of the most important mechanisms by which *Citrus* flavonoids exert their biological effects.

At a central level, other mechanisms involving SIRT1 accounted for the protective effects of flavonoids, mainly related to their antioxidant and anti-inflammatory properties. This is the case of NGN, well-known for its different biological effects [42], some of which are related to SIRT1 modulation. Indeed, a recent study, carried out both in vitro and in vivo, revealed that NGN protected from both brain function decline and dry age-related macular degeneration, by exerting SIRT1-mediated antioxidant effects [103,104]. Furthermore, the activation of SIRT1 by HES promoted the inhibition of NADPH oxidase-4 (NOX4) expression and protection against oxidative and inflammatory damage characterizing an in vivo model of neuropathy [105]. Remarkably, neurological dysfunctions also represent one of the most relevant age-related disorders. In this regard, Ahmad and co-workers observed that FIS was able to mitigate both inflammation and oxidative stress by modulating SIRT1/Nrf2 signaling pathways and suppressing the activated c-Jun N-terminal kinase (p-JNK)/NF-κB pathways in an in vivo model of age-related neurological disorders [106]. Finally, flavanone NAR might be considered as a therapeutic tool in the management of age-related disorders for its capability to counteract mitochondrial dysfunction in mice, through the activation of SIRT1 [107]. 

Interesting SIRT1-mediated anti-inflammatory mechanisms also concern the metabolic context. Several studies collected in a narrative review highlighted the beneficial effects of polyphenols on metabolic disease linked to their anti-inflammatory properties [40]. Indeed, the flavonoid nobiletin (NOB) was able to reduce liver inflammation and fibrosis through the suppression of the NOD-like receptor thermal protein domain-associated protein 3 (NLRP3) inflammasome in a SIRT1-dependent manner [108]. Liver inflammation was also mitigated by HSP, another relevant flavonoid found in *Citrus* fruits, exerting, similar to HES, antioxidant, anti-inflammatory, and anticancer properties, and also counteracting lung disorders [109,110,111]. In particular, HSP acted as a SIRT1 activator, which in turn led to the suppression of RelA/p65 acetylation, hampering NF-κB activation [112]. In the same context, Hua and co-workers proposed NGN as an activator of SIRT1 in the liver, leading to the improvement of non-alcoholic steatohepatitis (NASH). As a SIRT1 activator, NGN hindered hepatic inflammation and oxidative stress by promoting the deacetylation of liver kinase B1 (LKB1), PGC-1α, and NF-κB [113]. Interestingly, the same flavonoid was also able to prevent the pathogenesis of fibrotic disorders in vivo and in vitro through the regulation of signaling molecules, such as SIRT1, NF-κB, and ROS [114].

Moreover, it was recently reported that treatment with diosmin (DIO) against colitis counteracted colon oxidative damage and inflammation, through the upregulation of SIRT1 circular RNA (Circ-SIRT1) [115]. Also noteworthy is a study in which the association of HES and QUE increased SIRT1 levels in the liver and kidneys of diabetic rats, mitigating oxidative damage and hampering NF-κB activation [116]. Along this line, considering that one of the most validated methods to evaluate liver and kidney damage is the employment of lipopolysaccharides (LPSs), Rostami and collaborators demonstrated through this method the modulatory effect of MYR on SIRT1. Indeed, MYR reduced the serum levels of hepatic parameters, as well as the oxidative and inflammatory factors, through a mechanism characterized by the upregulation of hepatic SIRT1 [117]. Similarly, endotoxemic kidney injury was mitigated by RU in C57BL/6 mice by suppressing both oxidative and inflammatory processes via the activation of SIRT1 [118].

SIRT1-mediated antioxidant and anti-inflammatory activities were also responsible for cardio-protective effects induced by flavonoids in both in vitro and in vivo models. Along this line, LU as well as RU counteracted hypoxia/reoxygenation (H/R) in an in vitro model of myocardial injury [119] as well as in vivo myocardial ischemia/reperfusion (I/R) injury via activation of the SIRT1/NLRP3/NF-κB pathway [120]. Thanks to its antioxidant and anti-inflammatory properties, HSP and FIS showed cardio-protective effects by activating the SIRT1/Nrf2 signaling pathway [121,122].

In addition to the widely discussed antioxidant and anti-inflammatory effects, *Citrus* flavonoids were shown to play a relevant role by also hampering metabolic disorders through several other mechanisms, involving SIRT1 modulation. Overweight and obesity are becoming one of the major public health problems worldwide. Among the main comorbidities related to obesity are insulin resistance and type-2 diabetes (T2D), hypertension and cardiovascular disease (CVD), dyslipidemia, NAFLD, and renal dysfunctions [25]. A recent meta-analysis highlighted the beneficial effects of polyphenol supplementation on NAFLD [123]. Indeed, regarding disorders related to lipid metabolism, it has been demonstrated that NOB is capable of restoring the expression of SIRT1, blocked by high levels of free fatty acids in hepatocytes, thus reprogramming the altered circadian clock [124], as well as counteracting lipotoxicity in vitro [108]. On the other hand, NEO showed its therapeutic potential in the regulation of lipid metabolism by hampering lipogenesis in the liver and activating fatty acid oxidation thanks to the activation of SIRT1 [125]. The increase in SIRT1 mRNA expression was also observed in the brown adipose tissue of Swiss male mice treated with gallic acid (GA), leading to the improvement of body metabolism and glucose homeostasis [126]. In this latter context, Kaempferol 3-O-rutinoside (KOR) caused the overexpression of SIRT1, which in turn led to the upregulation of insulin-dependent phospho-insulin receptor substrate (p-IRS), protein kinase B (AKT), and AMPK signaling pathways, stimulating GLUT4 activation in vitro [127]. On the contrary, as regards liver dysfunction due to fat accumulation, Li and collaborators proposed kaempferol (KMF) as an activator of AMPK/SIRT1, in order to mitigate NAFLD [128]. Therefore, it appears that the activation of SIRT1 plays a pivotal role in the management of hepatic illnesses. Interestingly, Sayed and co-workers performed molecular docking simulations, suggesting that flavonoids are able to modulate SIRT1, eliciting pharmacologic activities in different hepatic diseases [129]. In this regard, both in vitro and in vivo studies promoted NAR as a modulator of SIRT1 activation, hindering pro-inflammatory, pro-oxidant, and pro-apoptotic signaling pathways [130].

Other biological effects of *Citrus* flavonoids mediated by SIRT1 modulation are exerted on endothelial dysfunction and cardiovascular aging [131]. In this context, NOB protected from myocardial I/R injury through the downregulation of miRNA-433 (miR-433), which favors SIRT1 upregulation [132], and it also protected against hepatic I/R injury by SIRT1/forkhead box O3a (FOXO3a) activation [133]. Again, the flavonoid NAR, isolated from immature dry fruits of *Citrus wilsonii*, was able to exert anti-apoptotic, anti-inflammatory, and antioxidant effects, attenuating the severity of myocardial I/R injury through SIRT1 activation [134]. The myocardial degenerative processes are often associated with senescence, and in this context, Testai and colleagues reported that NGN was able to target SIRT1, thus protecting against the myocardial degradative processes associated with senescence [135].

Finally, SIRT1 can be implied in tumorigenesis. In this process, an upregulation of SIRT1 was associated with the antiproliferative effect of NOB. Indeed, this flavone reduced the proliferation of nasopharyngeal carcinoma C666-1 cells, inducing apoptosis through the upregulation of the Poly ADP ribose polymerase (PARP-2)/SIRT1/AMPK axis [136]. Another interesting anticancer mechanism proposed for the employment of GA was the activation of the SIRT1/Nrf2 signaling pathways, which in turn led to the upregulation of the telomerase reverse transcriptase (*hTERT*) gene expression in HepG2 cells [137].

In Table 1, the evidence regarding the effects of flavonoids present in *Citrus* fruits through SIRT1 modulation is reported.

## 4. SIRT2

**Figure 5:**
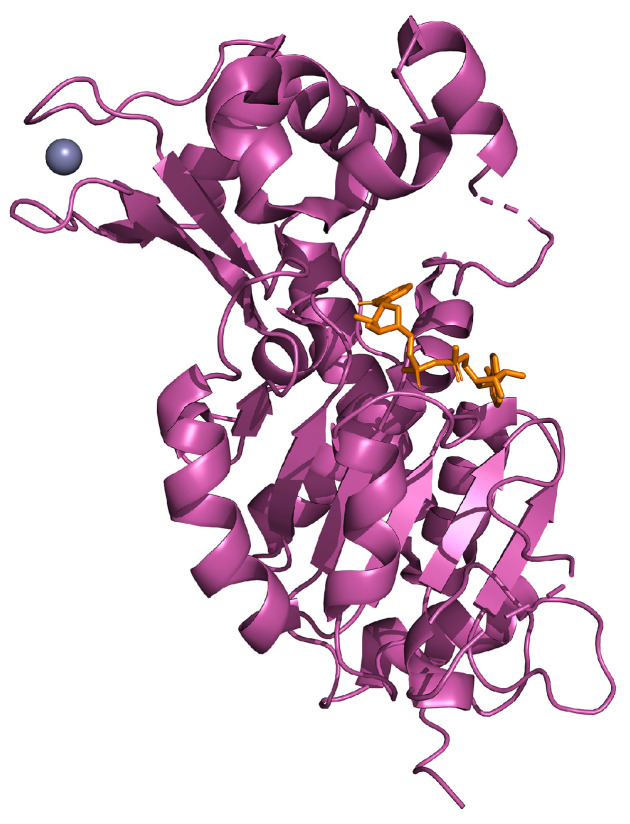
Human SIRT2 (in magenta) in complex with zinc (in violet) and NAD (in orange; PDB: 4RMG).

SIRT2 possesses a catalytic core of 304 amino acids and an N-terminal helical extension of 19 residues. The catalytic core is composed of an elongated pattern with two domains; the larger can be found in many different NAD(H)/NADP(H) binding enzymes, and the smaller domain contains a structural zinc atom. These two domains are separated by a large lipophilic area containing an active site for deacetylation of substrates [138] (Figure 5).

Given the multiple roles played by SIRT2 in regulating physiological and pathological signal transduction, it can be considered as a key target for the treatment of different illnesses [139], including neuroinflammation and Parkinson’s disease [140,141], as well as cancer [142] and CVD [143]. Interestingly, a QUE analogue derivative, 2-Chloro-1,4-naphtoquinone-quercetin, was able to hamper SIRT2 enzymatic activity by docking the substrate in the binding site [144], thus suggesting SIRT2 inhibition as a potential mechanism through which *Citrus* flavonoids exert biological effects.

It is well-known that neurodegenerative diseases, such as Parkinson’s, are associated with oxidative stress. In this regard, FIS was employed as a neuroprotective agent in a model of neuronal aging induced by oxidative stress and inflammation in rat brain. In particular, FIS exerted its neuroprotective effect by reducing pro-oxidant species and apoptotic cell death as well as ameliorating mitochondrial membrane depolarization in aging rat brain. The mechanism by which FIS exerted its effect is based on the upregulation of autophagy genes (*ATG3* and *BECN1*) and the downregulation of the *SIRT2* gene in aging brain [145]. In the same field, a recent study reported that treatment with ferulic acid (FA) causes the blocking of oxidative stress through ERK1/2-mediated activation of the Nrf2 and SIRT2 inhibition in an in vitro model of Parkinson’s disease [146].

In the context of tumorigenesis, Maugeri and colleagues reported the anticancer effect of BJe against hematologic malignancies, employing THP-1 monocytes as a model of acute myeloid leukemia. Indeed, BJe exerted its anticancer effect, resulting in a reduction in cell proliferation, blockage of the cell cycle in S-phase, and induction of apoptosis. This occurred because BJe inhibited SIRT2 activity and its gene expression, thus increasing the acetylation and then activity of p53. Finally, the reduced phosphorylation of AKT resulted in the link between SIRT2 and p53, suggesting the involvement of the SIRT2/AKT/p53 pathway underlying the anti-leukemic effects mediated by Bje [147]. More in depth, it was revealed that the flavanones present in Bje, namely NAR, HSP, NGN, and NEO, can block SIRT2 activity on the isolated recombinant enzyme, and the association of both NAG and HSP reduces THP-1 cell proliferation. Moreover, as observed in docking studies, these two flavanones bind the SIRT2 inhibitory site, acting as anti-leukemic agents [148]. 

It is noteworthy that, in contrast to other studies, Deng and co-workers revealed that limonin (LIM), a furanolactone belonging to the limonoid family, was able to exert protective effects against doxorubicin-induced cardiotoxicity through the activation of Nrf2 and SIRT2 signaling pathways [149].

Table 2 gathers the studies dealing with the role of *Citrus* flavonoid in modulating SIRT2.

## 5. SIRT3

**Figure 6:**
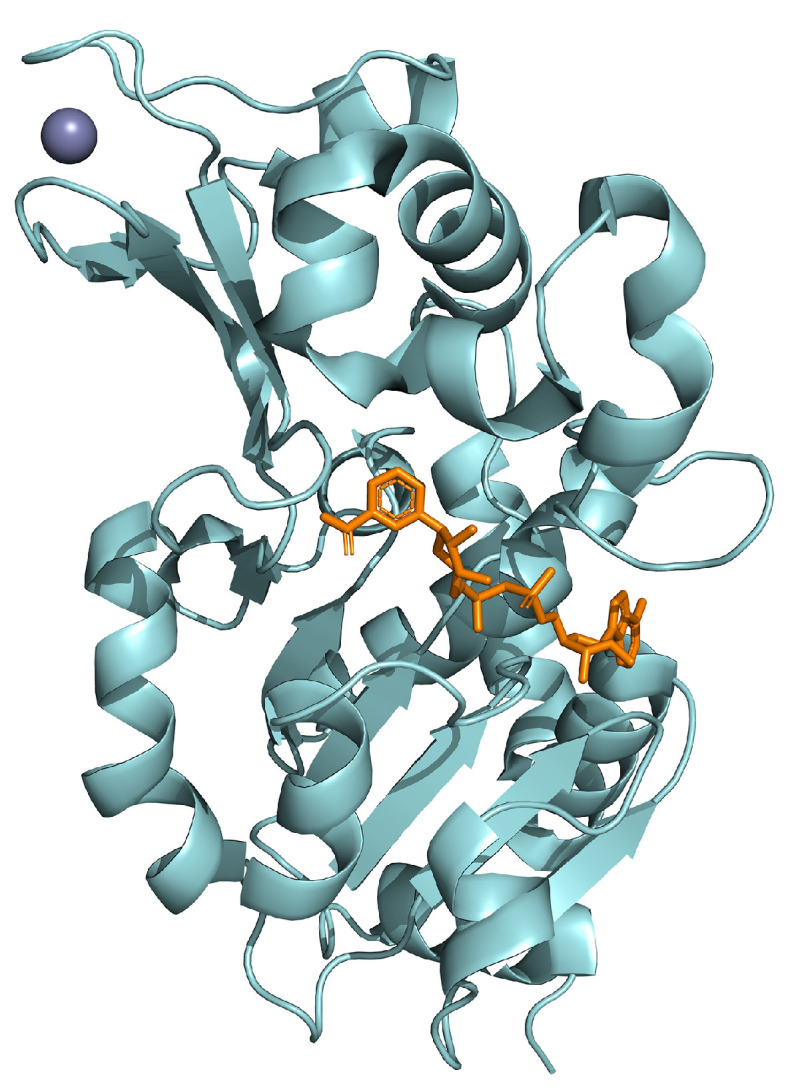
Crystal structure of SIRT3 (in cyan) in complex with zinc (in violet) and NAD (in orange; PDB: 4BV3).

SIRT3 is a NAD^+^-dependent deacetylase found mainly in mitochondria. SIRT3 is the only sirtuin affecting human lifespan, playing a key role in several mitochondrial metabolic processes such as oxidative stress and energy metabolism [150] (Figure 6).

Several studies highlighted the involvement of SIRT3 in neurodegenerative disorders [151], ischemic stroke, traumatic brain injury, intracerebral hemorrhage, neuro-inflammation along with heart failure, oxidative stress, autophagy, and apoptosis [152,153,154]. The beneficial effects of *Citrus* flavonoids on human health have been deeply investigated, representing important ingredients for nutraceuticals and functional foods [155]. 

Although it is well-known that obesity-related insulin resistance may be mitigated by shifting from a high-fat diet to a normo-caloric one [156], the supplementation of *Citrus* flavonoids such as MYR to the diet could be employed as a therapeutic tool against obesity, since it was demonstrated to favor the upregulation of SIRT3 expression in adipose tissue, improving mitochondrial metabolism in C57BL6/J mice [157]. Under hyperglycemic conditions, HSP, the aglycone form of HES present in peels of *Citrus* fruits, was shown to exert protective effects by counteracting LPS-induced secretion of pro-inflammatory cytokines in THP-1 macrophages. The mechanism underlying this anti-inflammatory effect included the blocking of TLR2/4, MyD88, and NF-κB phosphorylation through the upregulation of both SIRT3 and SIRT6 [158]. Furthermore, considering that hyperglycemic conditions lead to an increase in ROS production, an interesting study revealed that FIS and LU are able to hinder ROS production in high-glucose-treated THP-1 monocytes through the activation of SIRT1, SIRT3, SIRT6, and FOXO3a [159]. Remaining in the field of diabetic pathology, it has been reported that apigenin (API) improves renal injuries in both male Zucker lean (fa/+) rats (ZLRs) and male Zucker diabetic fatty (fa/fa) rats (ZDFRs) through the downregulation of NAD^+^-degrading enzyme CD38 and the increase in both intracellular NAD^+^/NADH ratio and SIRT3 [160]. 

At the hepatic level, Li and collaborators documented, in both in vitro and in vivo models, the anti-fibrotic effect of a monomer compound derived from HSP through the activation of AMPK/SIRT3, thus suggesting its employment as a hepatoprotective agent [161]. Similarly, this also occurred at the lung level, where baicalein (BAI) exerted its protective effect against fibrosis, regulating lung fibroblasts through an increase in SIRT3 expression [162]. 

However, oxidative stress and inflammation represent the main etiological causes of several pathologic conditions. Along this line, the antioxidant properties of *Citrus* flavonoids, even exerted through a modulation of SIRTs, led to beneficial effects in different ailments and oxidative disorders. NAR was able to fight mitochondrial oxidative stress in myocardial I/R-induced cardiomyocyte apoptosis through a mechanism involving the upregulation of the AMPK-SIRT3 axis [163]. Again, API exerted neuroprotective effects by increasing SIRT3 mitochondrial activity, reducing the accumulation of injured mitochondria, and promoting mitophagy [164]. Another two *Citrus* flavonoids, acacetin (ACA) and LU, were able to target SIRT3, promoting its upregulation, which in turn reduced the mitogen-activated protein kinase (MAPKs, p-38 and p-JNK) activation, by mitigating the oxidative damage and the skin photoaging caused by UVA and UVB, respectively, in both in vitro and in vivo experimental models [165,166]. 

Interestingly, SIRT3 was negatively associated with cancer. Consistently, Wang and colleagues reported that MYR-loaded nanoliposomes are able to inhibit cell survival in glioblastoma cells, through the downregulation of both SIRT3 and phosphorylated p53 [167].

In Table 3, the studies reporting the effects of *Citrus* flavonoids on SIRT3 are listed.

## 6. SIRT4

**Figure 7:**
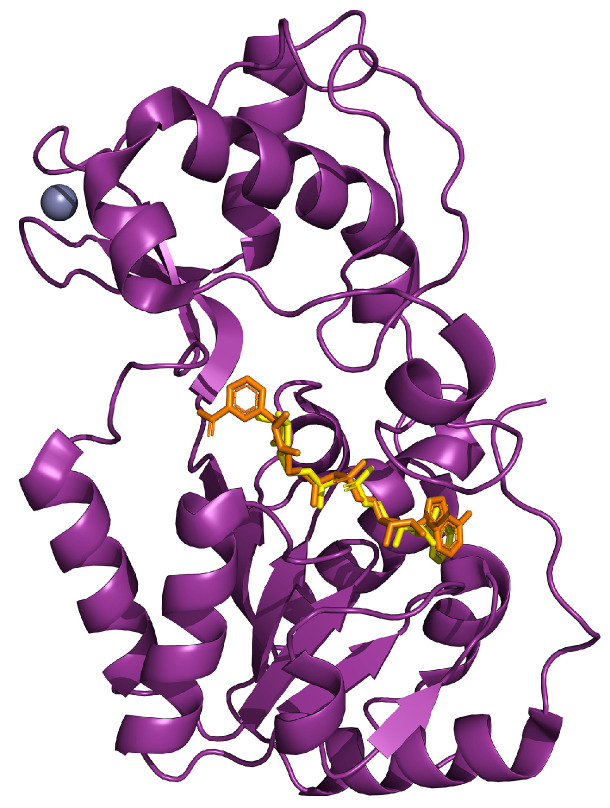
Crystal structure of SIRT4 (in purple) from *Xenopus tropicalis* in complex with ADP-ribose (in yellow) superimposed to NAD (in orange) and zinc (in violet; PDB: 5OJN).

Among the three mitochondrial SIRTs, SIRT4 has received the least focus from the scientific community [168]. Nevertheless, its key roles in both lipid and glutamine metabolism, as well as other possible enzymatic activities, have been reported [169]. The lack of studies is also reflected in the fact that the crystal structure of human SIRT4 is still missing, even though those of *Xenopus tropicalis* and *Danio rerio* possess a sequence similarity of the catalytic core of 67% and 65%, respectively [170] (Figure 7).

Along this line, the evidence on the role of natural molecules in SIRT4 is very limited. However, it has been reported that rhamnetin (RHM), one of the most abundant methyl esters in *Citrus* fruits, protected cardiomyoblasts against H_2_O_2_-induced cell death, also enhancing cell protection against redox imbalance. These effects were ascribed to a modulation of mitogen-activated protein kinases (MAPKs), which were upstream influenced by an induction of both SIRT3 and SIRT4 expression, thus supporting RHM cardio-protection (Table 4) [171].

## 7. SIRT5

**Figure 8:**
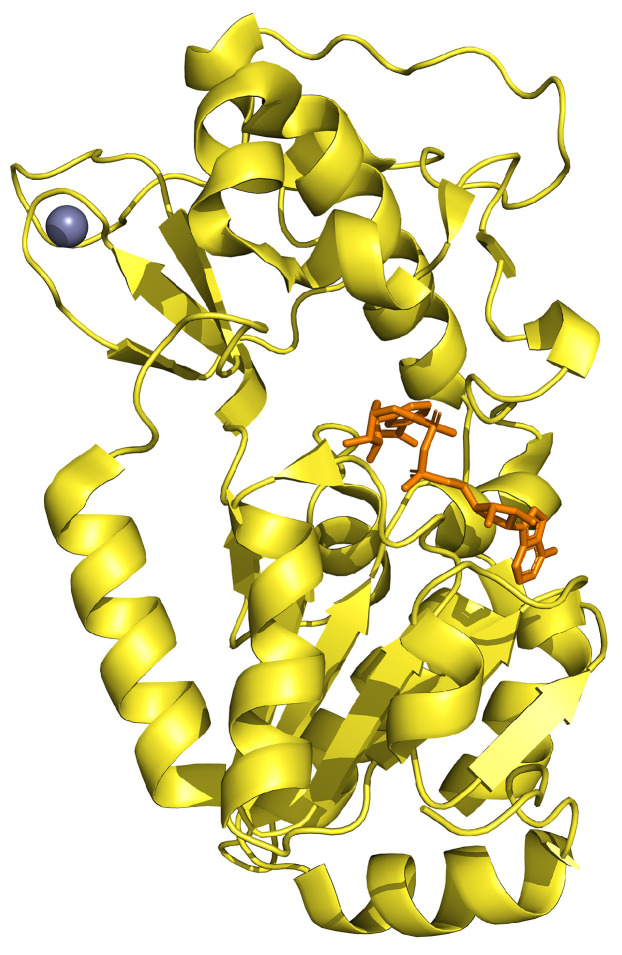
Crystal structure of SIRT5 (in yellow) in complex with zinc (in violet) and NAD (in orange; PDB: 3RIY).

SIRT5 is a NAD^+^-dependent deacetylase, containing both positively charged tyrosine and arginine in the active site, which are able to remove the acyl groups negatively charged from proteins [172]. SIRT5 consists of two domains; the larger one is a typical NAD^+^ binding site containing six parallel beta-strands (β1–3 and β7–9) forming a central sheet surrounded by several alpha-helices (α1, α2, α7, α10–13), while the smaller one is characterized by structural zinc ions and five α-helices (α3–5, α8–9) and three antiparallel β-sheets (β4–6) [173] (Figure 8).

SIRT5 is mainly localized into the mitochondrial matrix, playing a key role in the detoxification of ROS and in the regulation of protein substrates in fatty acid metabolism [174]. In this regard, recent studies revealed that SIRT5 is involved in metabolic diseases, particularly in hepatic steatosis [175], in cancer, and in SARS-CoV-2 infection [176]. Emerging evidence supports the capability of QUE to modulate SIRT5 expression, promoting the mitigation of several illnesses. In this frame, Chang and collaborators investigated the mechanism by which QUE counteracts myocardial fibrosis, improving cardiac function through an increase in SIRT5 expression, which in turn hampered oxidative stress and inflammatory response [177]. Furthermore, a recent study reported the capability of QUE to block DNA damage through the upregulation of SIRT5, which leads to the inhibition of PI3K/AKT phosphorylation, thus promoting apoptosis in an in vitro model of lung cancer [178]. In Table 5, the evidence on the effects of *Citrus* flavonoids on SIRT5 is reported.

## 8. SIRT6

**Figure 9:**
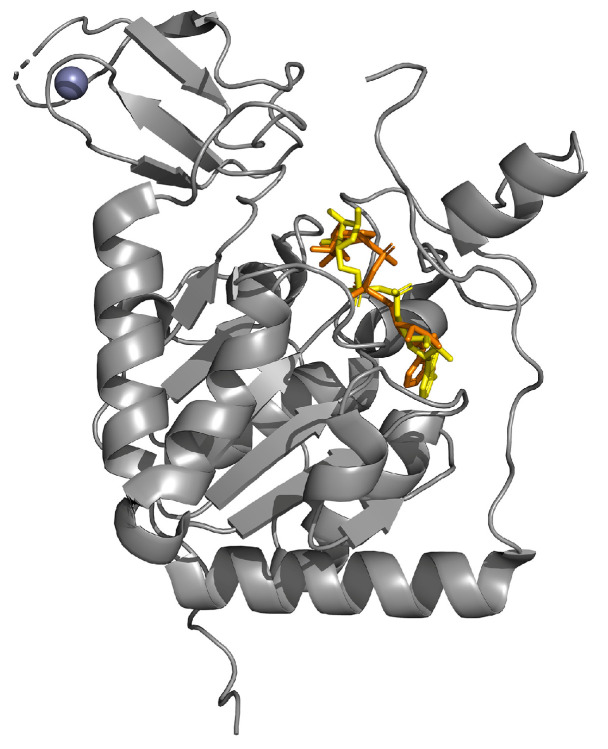
Crystal structure of SIRT6 (in gray) in complex with ADP-ribose (in yellow) superimposed to NAD (in orange) and zinc (in violet; PDB: 6QCD).

SIRT6 is a nuclear member of SIRT family formed by 355 amino acids, characterized by the typical core of about 250 amino acids, plus N-terminal extension, enzymatic core domain residues, and C-terminal extension [179]. In detail, SIRT6 is characterized by two domains. The large domain contains the nucleotide binding element as well as the Rossmann fold, which is elected for NAD^+^ binding. As regards the small domain, it is unique for SIRT6, containing Zn^2+^ binding loop able to stabilize the enzyme structure as well as the integrity of the catalytic domain [180] (Figure 9).

SIRT6 is able to promote long-chain fatty acid group deacetylation, as well as to catalyze the reaction of mono-ADP-ribosylation in chromatin silencing of the DNA repair proteins [181]. Moreover, SIRT6 acts as a signaling regulator of several illnesses, including cardiovascular diseases and diabetes mellitus, and it plays a pivotal role in the regulation of brain mitochondrial processes and in cancer [17,182,183,184,185]. Interestingly, through the employment of a screening method for the identification of novel SIRT modulators from plant extract, it has been observed that QUE is a candidate able to target SIRT6 [186].

Structurally, You and colleagues reported that QUE-based compounds activate as well as inhibit SIRT6 through the isoform-specific binding site for pyrrolo[1,2-a]quinoxalines, which can be considered as a versatile allosteric site for the modulation of SIRT6 [187]. Biologically, the activation of SIRT6 was associated with several beneficial effects. A very recent study investigated chalcone isoliquiritigenin (ISL), present in grapefruits, highlighting its capability to upregulate SIRT6, attenuating vascular endothelial cell pyroptosis mediated by NLRP3 [188].

Since SIRT6 plays a pivotal role in glucose and lipid metabolism, several studies focused on its modulation. In particular, it has been reported that HES is able to target and increase SIRT6 expression in THP-1 cells, mitigating diabetic inflammation, through the modulation of TLR/MyD88/NF-κB signaling pathways [158]. Interestingly, in the same in vitro model, Kim and co-workers suggested that LU and FIS inhibit high glucose-induced ROS production through the activation of SIRT1, SIRT3, SIRT6, and FOXO3a [159].

Therefore, the capability of *Citrus* flavonoids to counteract inflammatory processes was observed to occur through a modulation of SIRT6. Along this line, LU suppressed in vitro TNF-α-induced inflammatory injury and senescence via the SIRT6/NF-κB [189]. To support the well-known anti-inflammatory effects of HSP, Jing and co-workers suggested that it counteracts neuro-inflammation in vivo, by mitigating oxidative stress via SIRT6/NLRP3 in mice [190]. In the same field, the CAPE appeared to play a key role in neurological complication after anesthesia and surgery though a mechanism involving SIRT6/Nrf2 activation, reducing oxidative stress and favoring microglia-protective polarization [191].

On the contrary, the downregulation of hippocampal SIRT6 induced by FA counteracted depression-like behaviors by increasing the activity of AKT/collapsin response mediator protein 2 (CRMP2) signaling in mice [192].

Table 6 lists the studies which investigated the role of SIRT6 and *Citrus* flavonoids.

## 9. SIRT7

**Figure 10:**
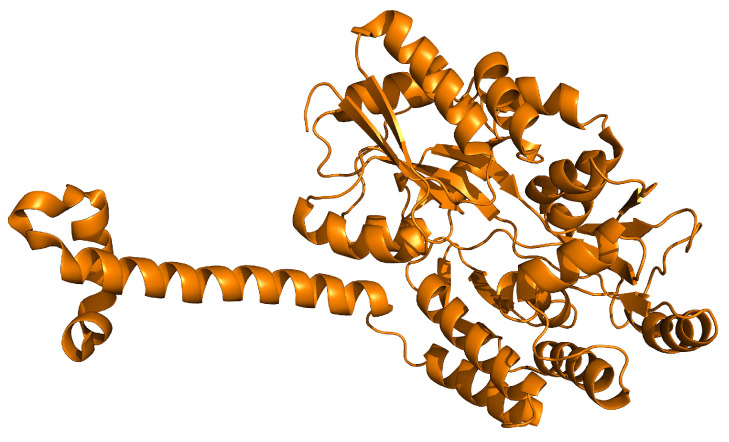
Crystal structure of N-terminal domain of human SIRT7 (PDB: 5IQZ).

SIRT7 is a NAD^+^-dependent histone deacetylase composed of 400 amino acids, showing deacetylase, desuccinylase, and deglutarylase activities [193]. SIRT7 contains a conserved catalytic core with long flanking N- and C-terminal extensions [194], and it has been reported that SIRT7 can be found in a chromatin-enriched fraction [195], despite the fact that a full crystal structure is still lacking (Figure 10).

It has been reported that SIRT7 plays a key role in the regulation of chronic inflammation [196], as well as in different kinds of cancer, including breast cancer [197], melanoma [198], and ovarian cancer [199]. As with the other SIRTs, *Citrus* flavonoids have been investigated for their effects on SIRT7. In this regard, it has been reported that HSP was able to target SIRT7, exerting a protective effect against calcific aortic valve disease both in vitro and in vivo. This effect was due to HSP’s capability to directly bind SIRT7, hampering the release of pro-inflammatory cytokines and ROS production, mitigating dysfunctional mitochondria, via the upregulation of Nrf2 [200].

Another study reported that FA, at low concentration, exerted neuroprotective effects and prevented neuronal apoptosis in H_2_O_2_-stressed PC12 cells. This is due to the stabilization and degradation of p53 through an increase in both *SIRT1* and *SIRT7* gene expression in vitro [201]. In Table 7, the studies reporting the effects of *Citrus* flavonoids on SIRT7 are reported.

## 10. Conclusions

**Figure 11:**
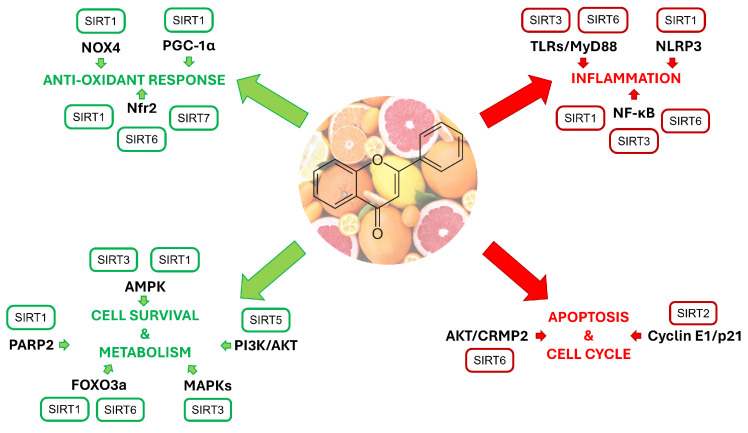
Pathways influenced by *Citrus* flavonoids via the modulation of SIRTs. Green arrows indicate activation, while red ones indicate inhibition.

SIRTs are NAD^+^-dependent deacetylases able to maintain cellular homeostasis by silencing genes and modulating the activity of different factors, thus unleashing cascades of numerous events when in action. Given also their widespread localization within cells (i.e., nucleus, cytoplasm, and mitochondria), it is not surprising that SIRTs are involved in several physio-pathological conditions. During recent decades, this has captured the interest of the scientific community, which has put forth great effort to unravel the true significance of SIRT regulation in cells. The multi-target capacity of natural products perfectly accords with the essence of SIRTs. Indeed, in this review, we highlighted the fact that *Citrus* flavonoids are able to elicit a wide plethora of biological effects via modulating the activity of SIRTs, acting as crossroads. Antioxidant, anti-inflammatory, hypolipidemic, and neuroprotective effects were exhibited by flavonoids through the activation of SIRT1, SIRT3, and SIRT6. On the contrary, the inhibition of SIRT2 was mainly associated with antiproliferative and neuroprotective effects. Beneficial effects, such as cardioprotective effects, were preliminarily observed from SIRT4 activation, and anti-inflammatory and anticancer effects were related to SIRT5 activity, while neuroprotective effects were mediated by SIRT7 (Figure 11).

Notably, the evidence of some members of the SIRT family is rich and robust, whereas others are still lacking studies to precisely define their role and hence investigate compounds able to target them. Considering that SIRTs belong to a family of multi-functional enzymes, a deepening of the current knowledge on the neglected SIRT members would be highly encouraged among scientists. Again, potential interactions on SIRTs or on more than one SIRT simultaneously should be considered, in order to fully define the mechanism of action and the selectivity rate of natural allies, such as *Citrus* flavonoids. Consequently, this could help to better establish their place in the management of several human illnesses.

To date, epigenetic inhibitors, such as histone deacetylase and DNA methyltransferase inhibitors, appear to represent an emerging scenario over conventional therapies, in different clinical settings. However, the impact of SIRT modulation on human health remains an open challenge among researchers. Adequate and well-established findings are essential for the development of effective therapies based on SIRTs. In this context, future investigations could pave the way towards combination therapies including natural and synthetic drugs or, even better, represent the starting point for the development of potent scaffolds targeting SIRTs.

Overall, this review helps in outlining a direction for further studies, thus suggesting *Citrus* flavonoids as holding potential promise in the development of novel effective drugs acting on the SIRT family.

## Figures and Tables

**Table 1:** Effects of *Citrus* flavonoids elicited via the modulation of SIRT1 in cells or animal models.

Experimental Models	*Citrus* Flavonoid	Effect	Reference
Wistar rats	HES	Antioxidant effect by activating miR-126-3p/miR-181a-SIRT1 network	[79]
Wistar albino rats	FIS	Reduction in testicular toxicity via SIRT1 activation	[80]
SD rats HUVEC cells	NGN	Antioxidant effect against endothelial damage by activating AMPKα/SIRT1	[81]
Swiss albino mice	NGN	Amelioration of chronic sleep deprivation-induced pain via SIRT1 activation	[82]
Primary rat NPMSCs	FIS	Anti-inflammatory and antioxidant effects mitigating intervertebral disc degeneration through the activation of SIRT1 pathway	[83]
Rat chondrocytes	RU	Mitigation of osteoarthritis pathogenesis through the activation of SIRT1	[84]
A549 cells	MYR	Anti-inflammatory effect via SIRT1/NF-κB pathway	[85]
C57BL/6 mice	HES	Anti-inflammatory and antioxidant effects by the upregulation of SIRT1/NF-κB	[90]
SD rats	NGN	Mitigation of polycystic ovary syndrome by upregulating the gut microbiota and SIRT-1/PGC-1α	[91]
SD rats	HES	Antioxidant and anti-inflammatory effects via SIRT1/FOXO activation in hepatic encephalopathy	[92]
NRK49F cells/in vivo	LU	Attenuation of renal anemia caused by renal fibrosis through the SIRT1/FOXO3 pathway	[93]
THP-1 cells	NGN, NAR, HSP, NEO	Anti-inflammatory effect through the modulation of AMPK/SIRT1 axis	[94,95]
Human NPCs	NGN	Anti-inflammatory effect through the activation of AMPK/SIRT1 axis	[96]
HepG2 cells	HSP	Anti-inflammatory effect by activating SIRT1-AMPK pathway	[97]
THP-1 cells/LDLR^−/−^ knockout mice	LU	Counteraction of atherosclerosis via the AMPK/SIRT1 signaling pathway	[98]
Rat NPCs	ORI	Antioxidant effect against mitochondrial dysfunction through AMPK/SIRT1 axis	[99]
BV2 cells/primary microglia	TAN	Anti-inflammatory effect via upregulation of SIRT1 in microglia	[100]
Kunming mice	CA	Neuroprotective effect via the activation of AMPK/SIRT1	[101]
PC12 cells	CAPE	Neuroprotective effect via the activation of AMPK/SIRT1	[102]
SD rats	QUE/NGN	Anti-inflammaging effect by increasing SIRT1 level in hippocampus	[103]
Kunming mice ARPE-19 cells	NGN	Antioxidant effect by upregulation of SIRT1	[104]
SD rats Rat glial C6 cells	HES	Antioxidant and anti-inflammatory effects by SIRT1/NOX4 activation	[105]
C57BL/6N mice	FIS	Protection from neuroinflammation by activation of SIRT1/Nrf2 axis	[106]
Swiss mice	NAR	Protection from mitochondrial dysfunction in lung by activation of SIRT1	[107]
AML-12 cells	NOB	Suppression of NLRP3 inflammasome by activating SIRT1	[108]
RAW264.7/AML-12—BALB/C mice	HSP	Protection from hepatic inflammation via AMPK/CREB through SIRT1 activation	[112]
ApoE^−/−^ mice AML-12 cells	NGN	Anti-inflammatory, antioxidant, antifibrotic effects in NAFLD/NASH by activating hepatic SIRT1	[113]
C57BL/6 mice/Caco-2 and IEC-6 cells	DIO	Amelioration of colon inflammation and oxidative stress via the circ-SIRT1/SIRT1 axis	[115]
Wistar rats	HES/QUE	Antioxidant effect by upregulating SIRT1, hampering NF-κB activation	[116]
C57BL/6 mice	MYR	Antioxidant and anti-inflammatory effects through the upregulation of hepatic SIRT1	[117]
C57BL/6 mice	RU	Alleviation of acute endotoxemic kidney injury by upregulating SIRT1	[118]
H9c2 cells	RU	Mitigation of H/R in myocardial injury increasing SIRT1 expression	[119]
SD rats	LU	Mitigation of myocardial ischemia reperfusion injury via SIRT1/NLRP3/NF-κB	[120]
Kunming mice	HSP	Antioxidant, anti-inflammatory effects in myocardial ischemia by activation of SIRT1/Nrf2	[121]
HepG2 cells/Primary hepatocytes from C57BL/6	NOB	Mitigation of lipid metabolism by upregulating SIRT1 in hepatocytes and circadian rhythms	[124]
HepG 2 cells/ Homozygous C57BL/6 (C57) mice	NEO	Reduction in lipid metabolism through AMPK/SIRT1/PGC-1α axis	[125]
L6 cells	KOR	Stimulation of glucose uptake through SIRT1 induction	[127]
HepG2 cells/db/db mice	KMF	Counteraction of NAFLD through the activation of SIRT1/AMPK axis	[128]
AML-12 cells	NAR	Protection from liver damage by upregulation of SIRT1	[130]
H9c2 myocardial cells	NOB	Protection against myocardial I/R injury via the modulation of the miR-433/SIRT1 axis	[132]
C57BL/6 mice	NOB	Protection against hepatic I/R via SIRT1/FOXO3a activation	[133]
SD rats	NAR	Attenuation of myocardial I/R by reducing oxidative stress and inflammation, through SIRT1 activation	[134]
Enzymatic assay, computational study, C57BL/6J, H9c2 cells	NGN	Anti-senescence effect by reducing inflammation and ROS enhancing the expression of SIRT1	[135]
C666-1 nasopharyngeal carcinoma cells	NOB	Antiproliferative effect by the upregulation of PARP-2/SIRT1/AMPK pathways	[136]

Caffeic acid (CA), caffeic acid phenethyl ester (CAPE), diosmetin (DIO), fisetin (FIS), gallic acid (GA), hesperetin (HSP), hesperidin (HES), human nucleous polposus cell (HNPc), human umbilical vein endothelial cell (HUVEC), kaempferol (KMF), kaempferol 3-O-rutinoside (KOR), luteolin (LU), myricetin (MYR), naringenin (NGN), naringin (NAR), neohesperidin (NEO), nobiletin (NOB), nucleous polposus cells (NPCs), orientin (ORI), quercetin (QUE), rutin (RU), Sprague-Dawley (SD), tangeretin (TAN).

**Table 2:** Effects of *Citrus* flavonoids after modulation of SIRT2 in cells or animal models.

Experimental Models	*Citrus* Flavonoid	Effect	Reference
Enzymatic assay	2-Chloro-1,4-naphtoquinone-quercetin	Potent inhibition of SIRT2 enzymatic activity	[144]
Wistar rat Primary neuronal cells	FIS	Neuroprotective effect against aging-induced oxidative stress by the downregulation of *SIRT2* gene	[145]
Enzymatic assay SH-SY5Y cells	FA	Antioxidant effect via the inhibition of SIRT2 activity	[146]
THP-1 cells	NGN/HSP	Anti-leukemic effect via reduction in SIRT2 activity	[148]

Ferulic acid (FA), fisetin (FIS), hesperetin (HSP), naringenin (NGN).

**Table 3:** Effects of *Citrus* flavonoids due to the modulation of SIRT3 in cells or animal models.

Experimental Models	*Citrus* Flavonoid	Effect	Reference
C3H10T1/2 cells/C57BL6/J mice	MYR	Anti-obesity effect through the upregulation of SIRT3 ex-pression in adipose tissue	[157]
THP-1 cells	HSP	Suppression of inflammation in diabetes via TLR/MyD88/NF-κB increasing SIRT3	[158]
THP-1 cells	FIS/LU	Suppression of oxidative stress in hyperglycemic condition through the upregulation of SIRT1, SIRT3, SIRT6	[159]
ZLRs and ZDFRs rats	API	Mitigates mitochondrial oxidative stress through the upregulation of SIRT3	[160]
LX-2 cells/C57BL/6J mice	HSP	Hepatoprotective effect by activating the AMPK/SIRT3 pathway	[161]
Mice	BAI	Counteraction of lung fibrosis by restoring SIRT3 expression	[162]
SD rats/H9c2 cells	NAG	Antioxidant effect in myocardial I/R through the activation of AMPK/SIRT3 axis	[163]
Swiss albino mice	API	Attenuation of neurotoxicity via promoting mitochondrial homeostasis by activating SIRT3	[164]
SD rats/Human dermal fibroblasts cells	LU	Protection from skin photoaging by upregulating the SIRT3/MAPKs axis	[165]
SD rats	ACA	Protection from skin photoaging by upregulating the SIRT3/MAPKs axis	[166]
DBTRG-05MG cells	MYR	Antiproliferative effects in glioblastoma cells by reducing SIRT3 levels	[167]

Acacetin (ACA), apigenin (API), baicalein (BAI), fisetin (FIS), hesperetin (HSP), luteolin (LU), myricetin (MYR), naringin (NAG).

**Table 4:** Effects of rhamnetin in modulating SIRT4 activity.

Experimental Models	*Citrus* Flavonoid	Effect	Reference
H9c2 cardiomyoblast cells	RHM	Cardioprotective and antioxidant effects due to an increase in both SIRT3 and SIRT4 expression	[171]

Rhamnetin (RHM).

**Table 5:** Effects of *Citrus* flavonoids due to the modulation of SIRT5 in cells or animal models.

Experimental Models	Citrus Flavonoid	Effect	Reference
HL-1 cells/C57BL/6J mice	QUE	Antioxidant and anti-inflammatory effects increasing SIRT5 expression	[177]
BEAS-2B, Human NSCLC, A559 and H1299 cells	QUE	Inhibition of DNA damage and induction of apoptosis via the direct binding and upregulation of SIRT5, along with the modulation of PI3K/AKT pathway	[178]

Quercetin (QUE).

**Table 6:** Effects of *Citrus* flavonoids due to the modulation of SIRT6 in cells or animal models.

Experimental Models	*Citrus* Flavonoid	Effect	Reference
THP-1 cells	HSP	Mitigation of diabetic inflammation through the modulation of TLR/MyD88/NF-κB signaling pathways, increasing SIRT6 expression	[158]
THP-1 cells	LU/FIS	Inhibition of ROS production through the elevation of SIRT6 and FOXO3a expression	[159]
Enzymatic assay/U2OS cells	QUE, LU	Modulation of SIRT6 activity	[187]
HUVEC cells	ISL	Decrease in vascular endothelial cell pyroptosis via the upregulation of SIRT6	[188]
HNPC cells	LU	Anti-inflammatory effect by upregulating SIRT6 and hindering the downstream activation of NF-κB pathway	[189]
C57BL/6J mice	HSP	Counteraction of neuroinflammation and oxidative stress by increasing SIRT6 levels	[190]
C57BL/6J mice/BV2 cells	CAPE	Mitigation of post-operative cognitive dysfunction, hindering oxidative stress by enhancing the SIRT6/Nrf2 pathway	[191]
C57BL/6 mice/Human HEK-293T and mouse HT-22 cells	FA	Reduction in depression-like behaviors by suppressing AKT/CRMP2 and acting as downregulator of SIRT6	[192]

Caffeic acid phenethyl ester (CAPE), ferulic acid (FA), fisetin (FIS), hesperetin (HSP), isoliquiritigenin (ISL), luteolin (LU), quercetin (QUE).

**Table 7:** Effects of *Citrus* flavonoids on the modulation of SIRT7 in cells or animal models.

Experimental Models	*Citrus* Flavonoid	Effect	Reference
C57BL/6 mice/ Docking studies/Human VICs	HSP	Protective effect in the aortic valve, increasing the SIRT7-mediated activation of the Nrf2–ARE axis	[200]
PC12 cells	FA	Neuroprotective effect through the upregulation of SIRT1 and SIRT7	[201]

Hesperetin (HSP), ferulic acid (FA).

## Data Availability

Not applicable.
